# Micro and Nanoplastic Contamination and Its Effects on Freshwater Mussels Caged in an Urban Area

**DOI:** 10.3390/jox13040048

**Published:** 2023-12-05

**Authors:** François Gagné, Eva Roubeau-Dumont, Chantale André, Joëlle Auclair

**Affiliations:** Aquatic Contaminants Research Division, Environment and Climate Change Canada, Montréal, QC H2Y 2E7, Canada; eva.roubeau@gmail.com (E.R.-D.); chantale.andre@ec.gc.ca (C.A.); joelle.auclair@ec.gc.ca (J.A.)

**Keywords:** *Elliptio complanata*, urban pollution, nanoplastics, microplastics oxidative stress, amyloids

## Abstract

Plastic-based contamination has become a major cause of concern as it pervades many environments such as air, water, sediments, and soils. This study sought to examine the presence of microplastics (MPs) and nanoplastics (NPs) in freshwater mussels placed at rainfall/street runoff overflows, downstream (15 km) of the city centre of Montréal, and 8 km downstream of a municipal effluent dispersion plume. MPs and NPs were determined using flow cytometry and size exclusion chromatography using fluorescence detection. Following 3 months of exposure during the summer season, mussels contained elevated amounts of both MPs and NPs. The rainfall overflow and downstream of the city centre were the most contaminated sites. Lipid peroxidation, metallothioneins, and protein aggregates (amyloids) were significantly increased at the most contaminated sites and were significantly correlated with NPs in tissues. Based on the levels of MPs and NPs in mussels exposed to municipal effluent, wastewater treatment plants appear to mitigate plastic contamination albeit not completely. In conclusion, the data support the hypothesis that mussels placed in urbanized areas are more contaminated by plastics, which are associated with oxidative damage. The highest responses observed at the overflow site suggest that tire wear and/or asphalt (road) erosion MPs/NPs represent important sources of contamination for the aquatic biota.

## 1. Introduction

Plastic pollution is found globally, raising concerns about its impacts on both humans and wildlife. Recent surveys revealed that worldwide plastic production reached 391 million metric tons in 2021 [1], and with inappropriate disposal regulations, it pervades soil and water ecosystems. Only 8.3% of total plastic production is recycled and 90% is produced from petroleum which contributes to the contamination problem. Canada and the European Union enforced a banning of single-use plastics except for water bottles and fishing gear which represents 70% of the marine litter. Polystyrene is a commonly used plastic polymer frequently found in packaging, building insulation, medical devices, and consumer products such as food and beverages. The omnipresence of this plastic results in the production of approximately 17 million tons of polystyrene waste annually [2]. Tire rubber, a derivative of petroleum products, represents a complex array of plastic-based compounds and is another important source of MPs and NPs in the environment [3,4]. Over 200 raw materials are included in tire composition, including elastomers, reinforcing fillers, plasticizers, and other chemical elements such as melamine, sulfur, trace metal elements (TMEs, mainly zinc), and polyesters. Tire wear particle (TWP) emissions were estimated at 1.12 million metric tons annually in the United States of America and represents 40 times the amounts of pesticides used in Germany. The entrance of tire wear particles in water bodies depends on the extent of collection and treatment of road runoffs, which is highly variable worldwide. Moreover, this contamination worsens from increased rainfall events in these times of climate change. Based on the above study, it is estimated, for a country the size of Germany, that 11,000 tons per year of TWP reach the rivers. Once released in the environment, plastics undergo five degradation pathways: hydrolysis, mechanical erosion, thermal and oxidative degradation, photodegradation, and biodegradation [5]. During these processes, large particles are degraded into microplastics (MPs; operationally defined between 5 mm and 1 µm) and into nanoplastics (NPs: between 1 and 1000 nm) in at least one of its dimensions. Moreover, the surface of these particles undergoes oxidation leading to the formation of hydroxylated (C-OH), carbonylated (C=O), and carboxylated (COOH) groups contributing to binding existing pollutants such as TMEs and organic chemicals (e.g., pesticides, aromatic hydrocarbons). NPs can cross cellular membranes, increasing their distribution in cells/tissues and initiate toxicity in aquatic organisms [6,7]. Recent surveys revealed that NPs from PS, polyethylene (PE), polyvinyl chloride (PVC), and polyethylene terephthalate (TEP) are found in the North Atlantic Gyre [8]. Although NPs were not investigated in this study, MPs were detected in large freshwater river systems such as the Saint-Lawrence River [9].

Most of the data on the toxicity of MPs and NPs are from single exposures of both marine and freshwater organisms [10,11]. In marine mussels, exposure to NPs leads to oxidative stress and lysosome membrane destabilization, leading to reduced growth and survival. In a comparative study of MPs and NPs uptake, the authors [12] found more of the 70 and 500 nm NPs than the 5, 10, and 100 µm MPs in the digestive gland of *Mytilus coruscus*. Nanoplastics were also found in the mantle tissues during depuration, suggesting translocation from the digestive tract to other tissues. Sustained oxidative stress could lead to tissue damage and degenerative disease, which are associated with aging or senescence [13]. Interestingly, concentrations of age-related pigments and lipofuscins were significantly increased by pollution in mussels exposed to urban pollution [12], although MPs and NPs were not readily assessed in tissues. These pigments are usually formed from the oxidation of proteins, lipids, and carbohydrates and are accumulated as plaques in cells and in tissues since the plaques do not degrade. This accumulation of plaque is also associated with degenerative diseases and involves inflammation in mammals [13]. Amyloids are usually formed in the stacking of β-sheets of amyloid-like proteins and are found in foot proteins and cement adhesives in mussels [14]. It would be of interest to understand the interplay between oxidative stress, damage (lipid peroxides or aldehyde formation on protein), and the formation of amyloids in sentinel mussel species for the environmental monitoring of water quality.

The purpose of this study was to determine the presence of MPs and NPs in tissues and explore their effects in freshwater mussels, *Elliptio complanate*, exposed to urban pollution. Experimentally caged mussels from a pristine lake were placed for 3 months downstream of a rainfall/road runoffs overflow, a large city (15 km), and a municipal effluent dispersion plume (8 km) to assess for the presence of MPs and NP uptake in mussels. Toxicological markers were based on the reported effects of NPs such as xenobiotic conjugation and oxidative stress with a specific emphasis on amyloid production. 

## 2. Materials and Methods

### 2.1. Mussel Caging Experiments

Materials in this study were purchased from Sigma Chemical company (Mississauga, ON, Canada) at the highest purity available. Uncoated polystyrene nanoparticles were purchased from Polyscience (Niles, IL, USA) at 20, 50, and 100 nm diameters. Freshwater mussels (*Elliptio complanata*) were sampled via scuba diving at a pristine lake completely isolated from anthropogenic activity located 100 km north of the city of Montreal in the Laurentians region (Québec, QC, Canada). Mussels were acclimated in 60 L aquariums (30–40 mussels each) for 20 days at 15 °C in UV-treated and charcoal-filtered tap water and fed 3–4 times per week with a commercial Coral reef feed (Phytoplex^®^, Gardenia, CA, USA) at 0.01% concentration. For in situ deployment, the mussels were caged in netting bags (1 m long × 0.25 m diameter, 1 cm diameter mesh, attached to a 1 kg cement block at 1 m depth from the shore). The bags were then placed at the following sites: (1) downstream of two rainfall overflow sites on the north shore of the Saint-Lawrence River (OVF: 45°38′26.3″ N; 73°29′15.6″ W; 45°36′05.2″ N; 73°30′33.6″ W), (2) downstream of the City of Montreal located 2 km upstream of a major municipal effluent dispersion plume (Downs-City: 45°39′28.5″ N; 73°28′37.9″ W), and (3) 8 km downstream in the municipal effluent dispersion plume (Down-effluent: 45°44′23.9″ N; 73°25′43.9″ W) at a depth of approximately 1 to 2 m, as explained earlier [15]. A reference site consisted of mussels held in cages in the laboratory during the exposure period. The mussels were exposed as such for 3 months (from July to October 2017). Water samples were also collected near the cages at the beginning, after 1.5 months, and at end of the exposure period for chemical analysis (pH, conductivity, total suspended solids, dissolved organic carbon, and ammonia concentrations). The cages were verified twice per month for mortality or cage loss. At the end of the exposure period, the mussels were collected, placed in ice-cold containers for transportation and depurated for 12 h in clean water to allow gut content purging. Mussels (10 mussels per cage and 3 cages per site, N = 30) were weighted, the shell length was determined, and the digestive gland was dissected on ice and homogenized in 5 volumes of ice-cold homogenization solution (50 mM NaCl, 10 mM Tris-acetate, 1 mM EDTA, 1 µg/mL apoprotinin, pH 8) with a steel polytron mixer (PD-Polytron PT 1300, 10,000 rpm 30 s on ice). The condition factor (CF) was determined by mussel wet weight (g)/longitudinal shell length (mm). A portion of the homogenate was centrifuged at 2500× *g* for 10 min at 2 °C. The supernatant (S2.5 fraction) was collected and stored at −85 °C until analysis.

### 2.2. Size Exclusion Chromatography of Polystyrene Nanoplastics in Tissues

The levels of polystyrene nanoplastics (PsNPs) were determined by size exclusion chromatography using the molecular rotor probe 9-(dicyanovinyl)-julolidine (DCVJ) [7,16] and a newly developed flow cytometric assay for larger MPs. The homogenates (250 μL) were extracted in 1 volume of saturated NaCl (5 M) and 1 volume of acetonitrile (ACN), mixed for 10 min, and centrifuged at 1500× *g* for 5 min to separate the ACN from the aqueous/saline phase. The ACN upper layer (200 μL) was mixed with 50 μL of 0.5 M NaCl in 0.2% Tween-20 and immediately applied to a Sephacryl S500 column (40 cm × 1 cm). This chromatography proved to be efficient for the separation of macromolecules reaching 150 nm. The elution buffer consisted of 1 mM NaCl containing 0.2% Tween-20 and a flow rate of 0.75 mL/min was selected and 1 mL fractions (in borosilicate glass tube) were collected for 32 mL total. For each sample, 50 μL of 100 μM DCVJ (in water) was added, and the fluorescence was measured (excitation 450 nm, emission 620 nm) as described above. The calibration of the instrument was achieved by using standard solutions of polystyrene NPs (50 nm diameter, Polyscience, Niles, IL, USA). The total volume (Vt) was obtained with NaCl conductivity peak and the non-polar low molecular weight components (lipids, metabolites) eluted at elution volume (Ve)/Vt between 0.9 and 1.1. The column was also calibrated with 20, 50, and 100 nm PsNPs (Polyscience, Niles, IL, USA). The DCVJ fluorescence data (relative fluorescence units at 450 nm excitation and 620 nm emission) were normalized to the total injected sample of the homogenate (total proteins). The relative levels of proteins in the S2.5 fractions were determined by measuring the absorbance at 280 nm of a 1/10 dilution of S2.5 fraction in 1 mM NaCl, 0.1 mM KH_2_PO_4_, pH 7.2 and 0.2% tween-20. Albumin was used for calibration.

### 2.3. Digestion via KOH and Determination of Microplastics via Flow Cytometry

The number of MPs in the size range of 3–20 µm were determined using flow cytometry based on autofluorescence of plastic polyethyelene and polystyrene microparticles [17]. The digestive gland homogenates were treated with 5 volumes of 10% KOH, incubated at 70 °C for 30 min, and left to stand at room temperature for 3–5 days until complete digestion of tissues. Samples were passed through a cellulose filter (0.22 µm pore size), rinced with 20–50 mL of PBS (140 mM NaCl, 1 mM KH_2_PO_4_, and 1 mM NaHCO_3_, pH 7.4) that had also been previously filtered through a 0.22 µm cellulose acetate filter (Thermo Fisher Scientific, Sainte-Foy, QC, Canada, 0.22 µm pore size). This step was carried out to remove traces of KOH and stabilize the pH at 7.4. The filter was then rinsed in 1 mL of 0.2% Tween-20 in water (by successive pipette aspirations) to remove the particles from the filters. The forward side scatter (size), side scatter, and fluorescence at 610 nm were recorded (488 nm excitation), as this wavelength showed a strong autofluorescence signal of PS commercial beads (3, 6, 10, and 20 µm diameter) used to calibrate the instrument. Procedural controls (methodological blanks) were realized using deionized water to account for the potential release of plastic particles during (1) the digestion process and (2) the filters rinsing step. The number of particles with positive fluorescence was used to determine the amounts of particles. Microplastic concentrations were expressed as particles between 3 and 20 µm/g tissue.

### 2.4. Measurements of Amyloids

The levels of amyloid proteins were determined using the Congo red assay [18,19]. Briefly, the 1500× g supernatant of the digestive gland homogenate was mixed with 1 volume of PBS and centrifuged at 10,000× *g* for 10 min at 20 °C. The supernatant was discarded, and the pellet resuspended in 1 mL of PBS. The pellet resuspension in PBS/centrifugation was repeated another 4 times. Then, the pellet was resuspended in 0.5 mL distilled water once and centrifuged again (10,000× *g* for 10 min at 20 °C). The pellet was resuspended in distilled water to liberate amyloids in the supernatant (soluble in water at low ionic strength). Amyloid determination was performed on the supernatant using Congo Red at 10 µM. Absorbance was measured at 490 nm using 10 µM Congo Red blank. As the presence of amyloids decreases the absorbance at 490 nm in a concentration-dependent manner [18], data were calculated as (1/A490 units)/mg proteins.

### 2.5. Determination of Protein Aggregation

The protein aggregation index in the S2.5 fraction was determined using a fluorescence methodology described elsewhere by [20,21]. A volume of 50 μL of S2.5 was added to 150 μL of 100 μM thioflavine T in PBS for 10 min. Fluorescence was measured at 400 nm excitation (485 nm emission) in dark microplates using the Synergy-4 fluorometer. Data were expressed as relative fluorescence units (RFU)/mg total proteins. 

### 2.6. Determination of Aldehydes

The levels of aldehydes were determined with a microplate fluorescence assay [22]. Briefly, 25 μL of S2.5 was added to 175 μL of 10 μM 4-aminofluorescein (in PBS) and incubated for 5 min at room temperature. Fluorescence (excitation 485 nm, emission 520 nm) was measured in borosilicate tubes (TBS-180, Turner Biosystems, Sunnyvale, CA, USA) and standardized with a fluorescein internal standard (1 μM). The data were expressed as fluorescence units/mg proteins.

### 2.7. Estimation of Lipid Peroxidation, Total Lipids, and Thiols

Levels of malondialdehydes, a proxy to evaluate the lipid peroxidation (LPO), were determined in the digestive gland by estimating the reaction product via fluorescence (540 nm excitation, 600 nm emission; Synergy-4, Biotek Instruments, Winooski, VE, USA) [23]. Tetramethoxypropane was used for calibration and data were expressed as μg of thiobarbituric acid reactants/mg proteins. The levels of total lipids were also determined in homogenates to assess the energetic status [24] by adding 180 μL of a Nile red solution (10 μM in PBS) to 20 μL of the S2.5 fraction and incubated for 5 min at room temperature. Measurements were made in terms of fluorescence (485 nm excitation/550 nm emission). Tween-20 (0.01%) was used as a standard and the data were expressed as relative fluorescence units/mg proteins. The levels of reduced thiols were determined using the dithiobisnitrobenzoic acid assay as previously described [25]. The absorbance was measured at 412 nm following the 15 min incubation with 0.1 mM substrate (in PBS adjusted to pH 8.0 with NaOH) in the S2.5 fraction. The data were expressed as absorbance increase/mg proteins.

### 2.8. Estimation of Metallothionein Content

The levels of metallothioneins (MT) in the S2.5 fraction were estimated using a silver saturation assay with cold detection of silver using atomic absorption spectrometry [26,27]. The S2.5 fraction was treated to 1 ppm silver (in 50 mM glycine-NaOH buffer, pH 8.5) for 15 min and 0.02% hemoglobin was added for 5 min. The mixture was heated at 100 °C for 2 min followed by centrifugation at 10,000× *g* for 5 min at room temperature. After another addition of 0.02% hemoglobin/heat denaturation/centrifugation at 10,000× *g* steps, silver concentration was determined in the supernatant via graphite furnace atomic absorption spectrometry equipped with Zeeman background correction and using silver nitrate for calibration and 1% ammonium phosphate as the matrix modifier. The data were expressed as µg MT equivalents/mg proteins. To estimate the levels of MT, it was considered that each mole of MT binds 12 moles of Ag for invertebrate MT.

### 2.9. Determination of Glutathione S-Transferase and Alcool Dehydrogenase Activity

Glutathione S-transferase (GST) activities were also determined in the S2.5 fraction in 96-well microplates as already described elsewhere [28]. The enzyme activity was expressed as a change in substrate/min/mg proteins in the digestive gland. The activity in alcohol/aldehyde dehydrogenase (ADH) was determined using ethanol and NAD+ as the co-substrates. Briefly, 25 µL of the S2.5 fraction of the homogenate was mixed with 0.1% ethanol and 100 µM NAD+ in PBS for 30 min at room temperature. Fluorescence readings for the formation of NADH were made at 350 nm excitation and 450 nm emission. The instrument was calibrated with 1 µM quinine sulfate for standardizing the excitation energy between assays. The data were expressed as NADH relative fluorescence units (RFU)/mg total proteins.

### 2.10. Data Analysis

The mussels (N = 30 per cage) of shell lengths between of 6 to 8 cm were used to minimize the influence of mussel size in the biomarker responses. The data (N = 10 mussels pe treatment) were subjected to rank analysis of variance (ANOVA) followed by the Conovan–Inman test to highlight significant changes relative to mussels caged for the same time period in the laboratory. Correlations between the variables were determined using the Pearson moment test with Bonferroni correction. Factorial analysis was performed using a principal component procedure to identify biomarker responses explaining most of the variance found between sites (Downs-city; downs-effluent; OVF) and to highlight the biomarkers with factorial scores > 70%. The level of significance was set at *p* ≤ 0.05. All tests were performed using the SYSTAT software (version 13, Palo Alto, CA, USA).

## 3. Results

### Nanoplastics and Microplastics in Mussels

**Figure 1:**
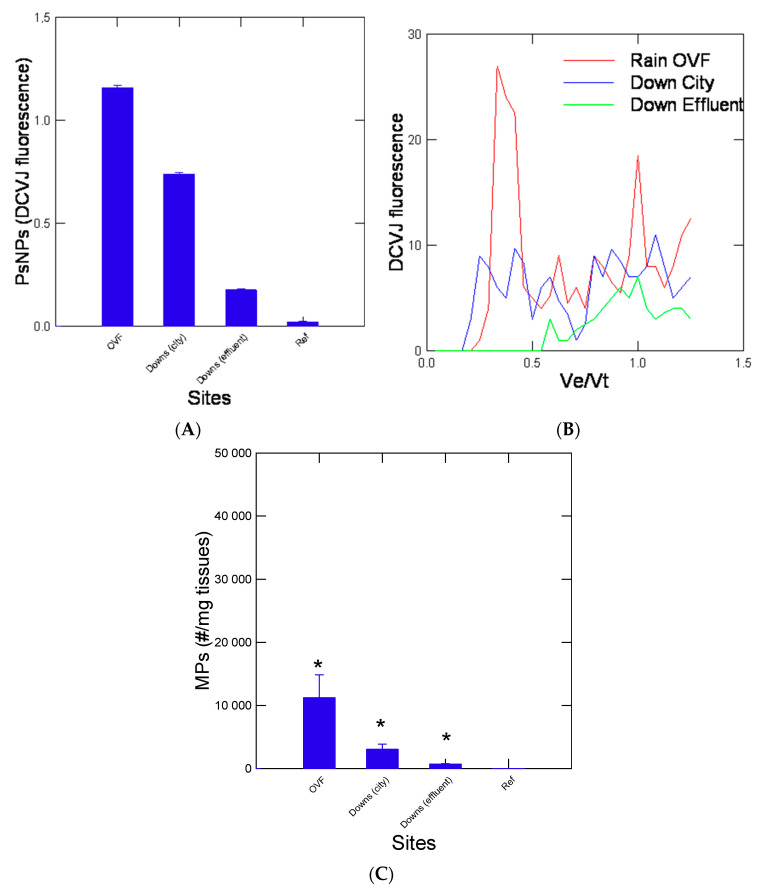
Size exclusion chromatography and flow cytometric assessment of plastic micro/nanoparticles in the digestive gland of mussels. Total levels of PsNPs (**A**), representative elution profiles (**B**) and MPs levels (**C**) were shown. The chromatography profile was obtained from 3 individuals at each site. The (bar graph) data represent the mean with standard error. The star symbol * indicates significance from the reference (Lake) site.

Freshwater mussels were collected at a pristine lake in the absence of human activity (habitation) and pollution sources. The levels in NPs were below the detection limit in digestive glands in mussels collected at the reference lake and caged in the laboratory for 3 months (Figure 1A). For all three caging sites, mussels revealed significantly higher NPs when compared to the reference site. Chromatograms of the ACN extracts of digestive gland homogenates revealed the presence of NPs in the following trend: OVF > Downs-City centre > Down-effluent > Lake (Figure 1B). The chromatograms revealed a complex distribution of DCVJ-positive peaks (plastic-like materials) with the appearance of large sizes at Ve/Vt = 0.3–0.4 (corresponding to the void volume of the column and NPs ≥ 100 nm based on the calibration with 100, 50, and 20 nm) especially for the OVF and Down the city centre mussels. For the downstream municipal effluent site, the DCVJ fluorescence peaks were lower and eluted at Ve/Vo > 0.5 suggesting smaller NPs compared to the other sites in the diameter range of 50–10 nm. For the quantitation of NPs, DCVJ fluorescence was taken in elution samples between Ve/Vt = 0.3 to 0.7 and normalized against the total proteins injected (280 nm absorbance). The data revealed that the OVF and the Downs City centre sites had elevated levels of NPs-like compounds compared to the municipal effluent site (Figure 1B). The determination of MPs in the 3–20 µm range revealed the same trends where the OVF sites contained the highest number of particles (Figure 1C). The levels of NPs in the digestive gland were correlated with the signal of MPs in the soft tissues (r = 0.85, *p* < 0.01). An analysis of covariance of NPs in digestive glands and sites using MPs in soft tissues as the covariate revealed the same differences across the sites suggesting that NPs was not only derived from MPs in tissues.

**Figure 2:**
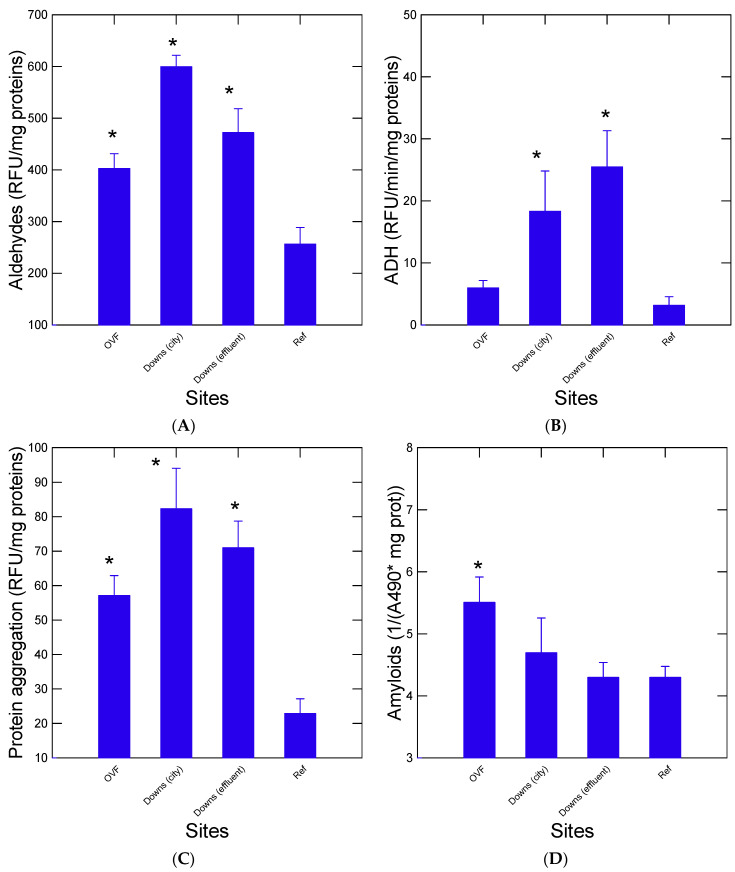
Change in protein properties and aldehydes in caged mussels. Proteins and aldehydes were determined by following changes in aldehydes (**A**), alcohol dehydrogenase (**B**), protein aggregation (**C**), and amyloids (**D**). The data represent the mean with the standard error. The star symbol * indicates significance with the reference site.

In addition to ADH activity for the oxidation of alcohols (hydroxylated products), changes in protein integrity were determined by measuring the levels of aldehydes, protein aggregation, and amyloids in digestive gland homogenates (Figure 2). The levels of aldehydes were significantly higher at all sites compared to the reference site and mussels exposed in Downs-city had the highest levels followed by the Downs-effluent and OVF (Figure 2A). Also, the activity of ADH was significantly elevated at Down (effluent) and at Down (city) compared to the reference site (Figure 2B). The ADH activity at the OVF was lower than the Down sites albeit correlated with aldehydes levels (r = 0.73, *p* < 0.001). A similar pattern for protein aggregation was observed for mussels of all three sites, in all of which the levels were higher than the reference lake (Figure 2C). Protein aggregation was also correlated with ADH activity (r = 0.65, *p* < 0.01) and aldehydes levels (r = 0.67, *p* < 0.01). The levels of amyloids were also determined in the digestive gland (Figure 2D). Significantly higher levels of amyloids were only found in mussels caged at OVF compared to the reference and the other sites (Figure 2D). Amyloids were significantly correlated with NPs in the digestive gland (r = 0.71, *p* < 0.01) and the protein aggregation index (r = 0.65, *p* < 0.01) but not with MPs.

**Figure 3:**
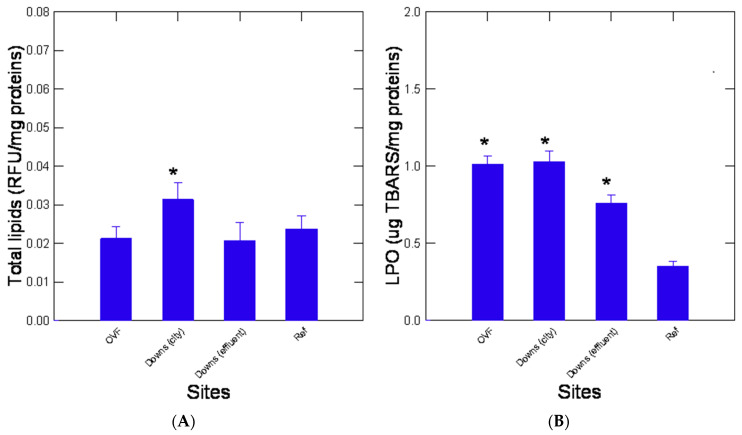
Lipid content and lipid peroxidation in caged mussels. Total lipids (**A**) and lipid peroxidation (**B**) were determined in the digestive gland of mussels. The data represent the mean with the standard error. The star symbol * indicates significance from the reference (Lake) site.

**Figure 4:**
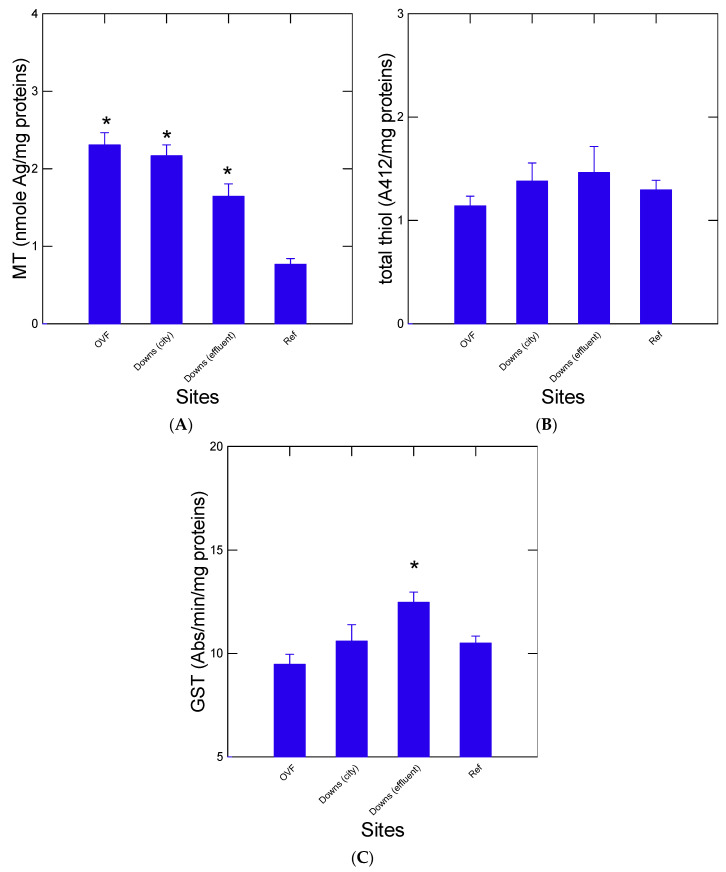
Thiol-based biomarker responses in mussels exposed to urban pollution. Thiol-based biomarkers were determined by measuring MT levels ((**A**): divalent metal and oxidative stress), total reduced thiol ((**B**): oxidative stress), and GST activity ((**C**): oxidative stress and phase II conjugation enzyme). The data represent the mean with the standard error. The star symbol * indicates significance with the reference site.

The impacts of urban pollution on lipid levels and integrity (LPO) were examined in the digestive gland of mussels (Figure 3). Lipid levels did not show drastic changes across sites. However, a significant increase at the OVF site was observed (Figure 3A). Lipid levels were also significantly negatively correlated with ADH activity (r = −0.64, *p* < 0.05). The levels of LPO were significantly increased at all sites compared to the reference site with the highest levels at the OVF and Downs city centre sites (Figure 3B). Correlation analysis revealed that LPO was significantly related with amyloids (r = 0.86, *p* < 0.001), protein aggregation (r = 0.72, *p* < 0.01), aldehydes (r = 0.63, *p* < 0.05), and, most notably, with NPs in the digestive gland (r = 0.9, *p* < 0.001) and the MP counts in tissues (r = 0.87, *p* < 0.001). This provides evidence that the most contaminated sites in terms of MPs and NPs involve oxidative stress and damage up to the formation of amyloids. However, this should be proven by more specific exposure experiments to NPs since mussels were exposed to many other urban pollutants. The effects of urban pollution on the levels of metal homeostasis, phase II biotransformation, and total thiols were determined in caged mussels (Figure 4). Metallothionein levels were readily increased at the urban sites with the highest levels found at OVF (Figure 4A), which were correlated with LPO (r = 0.79, *p* < 0.01), protein aggregation (r = 0.61, *p* < 0.05), and with both NPs (r = 0.82, *p* < 0.001) and MPs (r = 0.86, *p* < 0.001). The levels of thiols in the homogenates did not change across the sites (Figure 4B). The activity of the phase II conjugation enzyme GST was significantly increased in mussels downstream of the municipal effluent site (Figure 4C). Correlation analysis revealed that these two endpoints were not correlated with the other biomarkers nor with MPs and NPs.

**Figure 5:**
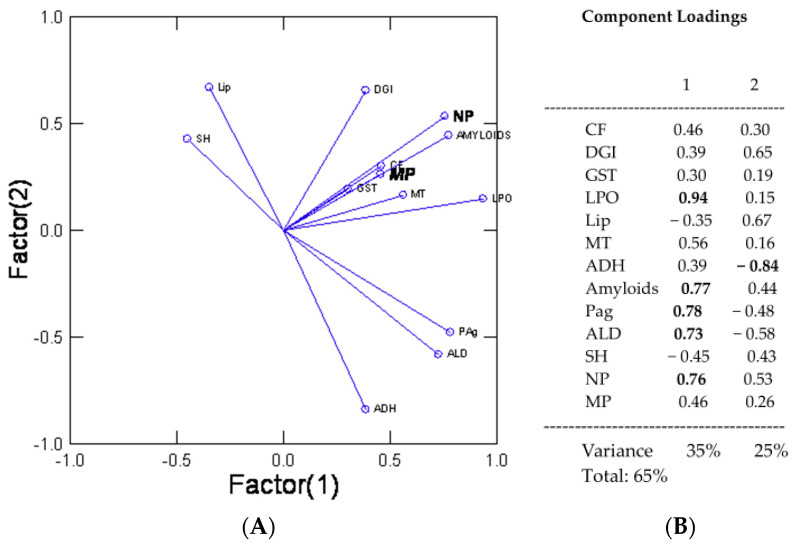
Factorial analyses of biomarker responses. Factorial analysis was performed to identify the endpoints that explained most of the observed variance in mussels exposed to overflow, downstream (city) and downstream (municipal effluent) and lake sites. The biomarkers with the highest factorial weights (>70%) were LPO, ADH, amyloids, prot agg, aldehydes, and NPs in the digestive gland. Abbreviations: condition factor (CF), digestive gland index (DGI), nanoplastics (NPs), microplastics (MPs), total thiol (SH), aldehydes (ALD), alcool/aldehyde dehydrogenase (ADH), metallothioneins (MT), protein aggregation (Pag), lipid peroxidation (LPO), lipids (Lip), and glutathione -S-transferase (GST). (**A**) Factor (1); (**B**) Component Loadings.

In an attempt to have a global understanding of the biomarkers’ responses in relation to the reported MP and NP levels in tissues in mixed pollution environments, a principal component analysis was performed (Figure 5). The analysis revealed that 60% of the variance was explained by two factors with the following prevalent biomarkers: NP levels in tissues, LPO, amyloids, digestive gland proteins, ADH, GST, CF, and DGI. The levels of NPs and MPs were closely related with amyloids, lipids, LPO, MT, thiols, digestive gland proteins, CF, and GST; however, LPO, lipids, and amyloids had the highest factorial weights (>70%).

## 4. Discussion

Overall, the levels of MPs in whole soft tissues and NPs in the digestive gland were higher in the following manner OVF > Downs city centre > municipal effluent after 3 months exposure. Following a 3-day exposure to monodispersed NPs (100 nm at 0.2 mg/L), 86.7% total NPs body weight was found in the digestive gland of the clam *Ruditapes philipppinarum* [29]. It was also reported, however, that other tissues could accumulate NPs albeit to reduced extend (gills: 5.2% > mantle: 5.1% > foot: 1.3% > exhalant siphon: 1.1% > adductor muscle: 0.6%). These results highlight the organotropism of NPs in marine bivalves and highlight the important bioconcentration that arises specifically in the digestive gland. Interestingly, a previous study revealed that 90 to 97% of MPs can be taken up by wastewater treatment plants [29]. In a second study, they showed that NPs and MPs are significantly lowered because of wastewater treatments when compared to the raw influent wastewater. In different municipal effluents, the authors measured removal efficiencies of 94 and 93% for NPs (from 12 to 0.71 µg/L) and MPs (from 26 to 1.8 µg/L), respectively [30]. In our study, we observed significantly higher levels of NPs and MPs in mussels exposed to all sites. However, these levels in Down (effluent) were not nearly as prominent as in OVF and Down (city), which could be at least partially explained by removal of NPs and MPs at the wastewater treatment plants. If that was the case, one could expect the resulting wastewater sludges to retain those plastics. Based on the chromatographic elution profiles with 50 nm PsNPs standards in the present study, the number of NPs in the 10–150 nm size range was in the order of 2.1 × 10^5^ nanoparticles/mg tissues at the most contaminated OVF site. Based on the volume of a sphere (V = 3/4πr^3^), a 3 µm diameter MPs volume contains the equivalent of 2.16 × 10^5^ particles for a 50 nm diameter. If this holds true, then the number of 50 nm NPs in mussels should be 2.38 × 10^9^ particles based on the levels of MPs in tissues, assuming of course that the particles are mostly spherical. It follows that the digestive gland of mussels contains 4000 fewer NPs than expected based on MPs levels and considering differences in the digestive gland vs. visceral (digestive gland and gonad tissues) masses. This suggests that NPs do not originate solely from MPs (if so, their levels would be negatively correlated with each other and at the corresponding amount) but rather are exposed to a mixture of MPs and NPs at the OVF site. Recent studies suggest that NPs are less bioavailable (retained) than MPs in mussels despite their ability to penetrate cells [31]. In the freshwater mussel *Dreissena bugensis*, the concentrations of 200 and 1000 nm NPs were less than the exposure concentration. In the same study, the particles were poorly rejected, taking up to 20 days to eliminate, and there was no significant difference between the tissue levels of NPs of 200 and 1000 nm at 1 pM concentration, approximately 5 × 10^8^ particles/mussel or in the order 2 × 10^6^ particles/mg soft tissues, which was 10 times higher than the reported concentrations of NPs in the present study (2.3 × 10^5^ NPs/mg tissues). 

In the context of OVF resulting from rainfall combined with sewer overflows, if emissions of combined sewers occur within 20 days then this could lead to the accumulation of NPs in mussels. This is especially the case for municipal effluents, which are continuously released in the environment. Based on these results, it appears that the bioaccumulation of NPs is relatively low, perhaps lower than the exposure concentrations in water, as was previously observed for PsNPs [32]. A lower accumulation for 70 nm diameter NPs but not for 10 µm diameter MPs was found in the absence algae (food intake) using filtration [33]. This suggests that MP/NP accumulation is size-dependent, where larger particles (in the same range of phytoplankton during feeding), would be preferentially taken up, finding their way into the digestive system. It is also possible that mussels exposed to foreign materials/contaminants reduce filtration during the episodic release of street runoff/sewer overflow. In *Corbicula fluminea* clams, exposure to 80 nm NPs and 6 um MPs reduced filtration rates and acetylcholinesterase activity and either the absence or presence of ciprofloxacin, an antibiotic found in municipal effluents [34]. Ciprofloxacin toxicity was reduced in the presence of NPs and MPs perhaps by reduced siphoning rates for the NPs–antibiotic treatment group. Hence, the reduced levels of MPs/NPs in mussels exposed to municipal effluents could result from (1) their reduced concentrations in wastewaters (plastics would be retained at the wastewater treatment plant), (2) reduced filtration rates, and (3) particle size selection by mussels.

It is worth highlighting that tissue levels of NPs were significantly correlated with LPO, MT, and amyloids, as with MPs, except for amyloids. Amyloids are considered a final consequence from β-sheeted protein denaturation and aggregation during prolonged oxidative stress, which is associated with cell aging and senescence. An analysis of covariance was performed on LPO levels using the levels of NPs in tissues to statistically tease out the contribution of NPs towards oxidative stress in a mixed pollution context. The analysis revealed that LPO levels at the highest contaminated site OVF in NPs were no longer significantly higher than the reference site, suggesting that NPs contributed significantly to LPO at the OVF site. This was supported in clams that had elevated levels of the antioxidant enzyme catalase and LPO, as determined using the malonaldehyde assay when exposed to 70 nm NPs and 10 µm MPs [34]. In another study with *Hydra vulgaris* exposed to NPs, both 50 and 100 nm NPs led to increased LPO in addition to anisotropic changes (via fluorescence polarization) indicative of nematic liquid crystal formation in the cytoplasm [21].

The increased MT concentrations in the digestive glands of mussels caged at OVF, Down (city), and Down (effluent) is a typical response to metal contamination and also to oxidative stress (e.g., MT can be also induced by oxidants). Tire wear particles are thought to pervade many environments near roads and should be present in combined sewer overflows as well [35]. Tire wear from street runoff also generates various compounds such as tire wear nanoparticles, which are known to be rich in sulfur and zinc, and copper [3]. Given that MT induction involves free Zn, Cu or other divalent metals, this suggests that the zinc associated with tire wear is labile and perhaps released from weathering involving oxidation. Indeed, tire wear NPs leachates contain up to 3 mg/L zinc with a mean particle diameter of 100 nm [3]. The study also showed that about 3% of the total levels of zinc in the leachates were associated with the nanoparticle fraction (between 200 nm and 20 kDa particles). In addition, plastic particles (not only those derived from tire wear) may act as carriers for several contaminants such as heavy metals (Ni, Zn, Cd, and Pb), polyaromatic hydrocarbons, and pesticides [36,37]. In the present study, MT levels were highest at the rainfall/street runoff overflow sites, which were expected to contain tire and asphalt wear NPs and metals. This is in keeping with the highest levels of MT at sites with high NPs and MPs in tissues (overflow and downstream, the city centre sites). 

With respect to GST activity, a major organic xenobiotic conjugation enzyme, its activity was significantly increased at the municipal effluent plume, which are well known emitters of many organic compounds of human and industrial origins (pharmaceutical and personnel care products, polyaromatic hydrocarbons, etc.). Indeed, municipal effluents are well-known sources of various organic pollutants and induce GST activity in mussels [38]. The activity of ADH was also examined as a potential biomarker of plastic exposure in this in situ biomonitoring study [39]. Indeed, aldehydes and alcohols forming on NPs during weathering [40] could be theoretically transformed into carboxylates by this enzyme. Indeed, its activity was increased at both urban sites compared to the reference site but was the highest at the municipal effluent site, suggesting that other contaminants were at play. This was corroborated by increased activity of GST at the municipal effluent site. ADH activity in mussels downstream of the overflow site was not altered when compared to the reference site. One possible explanation for this is the inhibition of ADH activity by tire wear zinc- and sulfur-rich NPs [40]. ADH is a zinc-containing enzyme, and its activity could be inhibited by zinc chelators (sulfur compounds) and zinc-mimicking metals such as copper, cadmium, and nickel [41,42]. This is consistent with the highest levels of MT at the OVF site, which lacks ADH induction. This suggests that ADH could be inhibited by zinc chelators, such as sulfur compounds, and metal competitors, such as Cd [43,44], which abound in tire- and asphalt-wear particles in road/street runoff and overflow sites in urban areas.

## 5. Conclusions

This study sought to examine the presence and potential effects of MPs and NPs in the freshwater mussel *E. complanata,* caged at various sites in the Saint-Lawrence River (Québec, Canada). The data revealed that mussels accumulated both MPs and NPs in all three examined sites in the following order: OVF > Down (city) > Down (effluent). This is consistent with the reported ability of wastewater treatment plants to mitigate plastic pollution. The presence of MPs and NPs was associated with oxidative damage leading to LPO and protein degradation (amyloidosis) in mussels. By investigating the associations between plastic exposure and the physiological effects of caged mussels, this study (1) shows that freshwater mussels downstream of large urban areas are exposed to plastic contamination and (2) strengthens the hypothesis that *in situ* plastic contamination elicits deleterious physiological changes in *E. complanata*. Furthermore, this study reinforces the idea of using *E. complanata* mussels as bioindicator species for environmental biomonitoring studies involving municipal effluents and plastic contamination.

## Data Availability

The data presented in this study are available on request from the corresponding author.
